# The Possible Role of Microbial Proteases in Facilitating SARS-CoV-2 Brain Invasion

**DOI:** 10.3390/biology10100966

**Published:** 2021-09-27

**Authors:** Nozethu Mjokane, Olufemi S. Folorunso, Adepemi O. Ogundeji, Olihile M. Sebolai

**Affiliations:** Department of Microbiology and Biochemistry, University of the Free State, 205 Nelson Mandela Drive, Park West, Bloemfontein 9301, South Africa; nmjokane@gmail.com (N.M.); foxxyphemmzy@gmail.com (O.S.F.); ogundejiao@ufs.ac.za (A.O.O.)

**Keywords:** *Cryptococcus*, co-infection, neurotropism, protease, SARS-CoV-2

## Abstract

**Simple Summary:**

There is limited information on how SARS-CoV-2 reaches the brain. Thus, this paper examines how, during co-infection, a protease-secreting microbe may facilitate brain invasion by SARS-CoV-2. An understanding of this potential invasion mechanism could lead to better SARS-CoV-2 intervention measures.

**Abstract:**

SARS-CoV-2 has been shown to display proclivity towards organs bearing angiotensin-converting enzyme (ACE2) expression cells. Of interest herein is the ability of the virus to exhibit neurotropism. However, there is limited information on how this virus invades the brain. With this contribution, we explore how, in the context of a microbial co-infection using a cryptococcal co-infection as a model, SARS-CoV-2 could reach the brain. We theorise that the secretion of proteases by disseminated fungal cells might also activate the S2 domain of the viral spike glycoprotein for membrane fusion with brain endothelial cells leading to endocytosis. Understanding this potential invasion mechanism could lead to better SARS-CoV-2 intervention measures, which may also be applicable in instances of co-infection, especially with protease-secreting pathogens.

## 1. Introduction

The respiratory failure caused by the novel coronavirus was first reported in December 2019 in Wuhan, China [1]. Today, this novel virus has been classified as a severe acute respiratory syndrome coronavirus-2 (SARS-CoV-2; commonly known as COVID-19). The virus targets the human pulmonary system with frequent detection in the nasopharynges swab (sensitive) and saliva (more sensitive) either with antibody–antigen immunoassay or real-time polymerase chain reaction (RT-PCR) diagnosis [2,3,4,5,6,7]. When inhaled, the virus could reach the lung space and inflame the alveoli, increasing levels of pro-inflammatory cytokines—leading to the infiltration of mononuclear cells (such as lymphocytes, monocytes, and macrophages) and accumulation of fluid in the lungs [8]. Further work showed that signalling pathways involving G-protein coupled receptors (GPCR) are usually arrested during pulmonary SARS-CoV-2 infection. This leads to perturbation of epithelial transport processes and homeostasis surface secretion involving cystic fibrosis transmembrane conductance regulator (CFCR) protein kinase A coupled Cl^−^ channel and epithelial Na^+^ channel [9]. We speculate that accumulated fluid from incontinence secretion from pneumocytes may be due to epithelial surfactant degradation and dysfunctional endothelial cells from impaired nitric oxide bioavailability caused by this infection. This consequently results in a pneumonialike disease with difficulties in gas exchange, making it hard to receive enough oxygen and expel carbon dioxide. The symptoms of pneumonia can range from mild to severe, including cough, loss of appetite, shortness of breath, fever, and chest pain [10,11,12]. However, severe cases can lead to acute respiratory distress syndrome (ARDS) and, subsequently, multiple organ failure in the lungs, heart, brain, kidney, and others [13,14,15,16].

SARS-CoV-2 is the current seventh member of the coronaviridae family (genus: β-coronavirus), exclusively infecting humans [1], but some wild animals are considered to be the carriers of this virus, especially bats [17] and Malayan pangolins [18,19]. Inarguably, SARS-CoV-2 infection is generally acquired from a contaminated environment, and it is rapidly transmitted via person-to-person contact [16,20,21,22]. SARS-CoV-2 is assigned a basic reproduction rate between 2.2–2.5 [21,23], which means every potential carrier, usually asymptomatic/atypical symptomatic, could have spread the infection to more than two people in their immediate environment. Significantly, SARS-CoV-2 transmission is driven by the family cluster more than the community spread [24].

A large proportion of the mortality comes from patients with underlying diseases. By assessing the risk factors associated with adult COVID-19 patients in various studies, hypertension, diabetes, chronic obstructive pulmonary disease (COPD), cardiovascular or cerebrovascular disease, hepatic dysfunction, obesity, renal failure, and cancer have become the dominant comorbidities with the highest mortality rate [16,25,26,27,28,29,30,31,32]. An interesting disease aspect is the manifestation of SARS-CoV-2 pneumonia in the presence of other invasive, pneumonia-causing bacteria and fungi. However, research into the existence of such co-infections lags far behind that of non-infectious diseases (Table 1). In Jiangsu, China, early assessment of COVID-19 patients for microbial co-infections (i.e., within one to four days of SARS-CoV-2 infection) showed that over 90% of such patients were infected with other respiratory pathogens, more so bacterial pathogens [33]. More importantly, the proportion of mixed co-infections was also confirmed to increase with SARS-CoV-2 severity. The above study is critical as it shows how such co-infections may hamper the vaccine response.

Therefore, there is an urgent need to research how SARS-CoV-2 pneumonia differs from other types of pneumonia. Moreover, such studies could potentially assist in understanding how SARS-CoV-2 may disseminate to invade other organ systems. Herein, we speculated that patients infected with SARS-CoV-2 would also be at a greater risk of co-developing the opportunistic cryptococcal pulmonary infection. More importantly, we use a cryptococcal infection as a possible model to explain how SARS-CoV-2 could invade the brain. This organism is endowed with a pool of extracellular hydrolytic proteases that may modulate SARS-CoV-2 proteolytic activation or degrading the cellular gap junction extracellular matrix to promote transcellular migration. One such example is the action of cryptococcal serine proteases, metalloproteases and aspartyl peptidases, capable of compromising the cell gap junction and enhancing the intracranial invasion of viral particles. In this paper, we examined the pathogenesis of SARS-CoV-2 and proposed a possible role of *C. neoformans* serine proteases, metalloproteases (the fungalysin, Mpr1) and aspartyl peptidases (Major aspartyl peptidase 1, May1) in enhancing SARS-CoV-2 brain invasion. We, thus, re-iterated the need to consider the microbial co-infection with SARS-CoV-2 in the management of the COVID-19 pandemic.

## 2. SARS-CoV-2: Current Understanding of Its Possible Brain Invasion

The route of viral dissemination into the brain region remains an enigma; however, neurological dysfunctions associated with brain and central nervous system (CNS) invasion by this virus has been emphasised [34,35,36,37,38,39,40]. In the earlier report of SARS-CoV-1 brain invasion in human angiotensin-converting enzyme (hACE2) transgenic mice, brain invasion occurred via the olfactory bulb with a rapid infection that spreads transneuronally, resulting in a significant neuropathy in the cardiorespiratory region of the medulla, causing death [41]. This report demonstrates the significance of ACE in the neuronal and glial cells viral invasion in the human CNS. For this reason, it has been speculated that SARS-CoV-2 may invade the human brain parenchyma, causing neuronal dysfunction; however, little is known about how SARS-CoV-2 reaches the brain. The extrapulmonary dissemination of SARS-CoV-2 via the blood and lymphatic vessels is established, and the tendency to invade and infect other organs of the human body is very high. This is because almost all the vital organs of the body possess ACE2-expressing cells, including the vascular endothelial cells and arterial smooth muscle cells [42]. A few investigations on the systemic spreading and organ invasion of SARS-CoV-2 have shown that this virus may spread into the brain via hematogenous dissemination, synaptic end junction via a neural pathway, and retrograde/anterograde neurones [41,43,44,45]. The neuropathic effect of SARS-CoV-2 is caused via the invasion of the meningeal endothelial layer within the blood–brain barrier (BBB), which makes the virus associate with the ACE2 receptor-expressing neuroglial cells in the medulla region of the brain.

**Figure 1:**
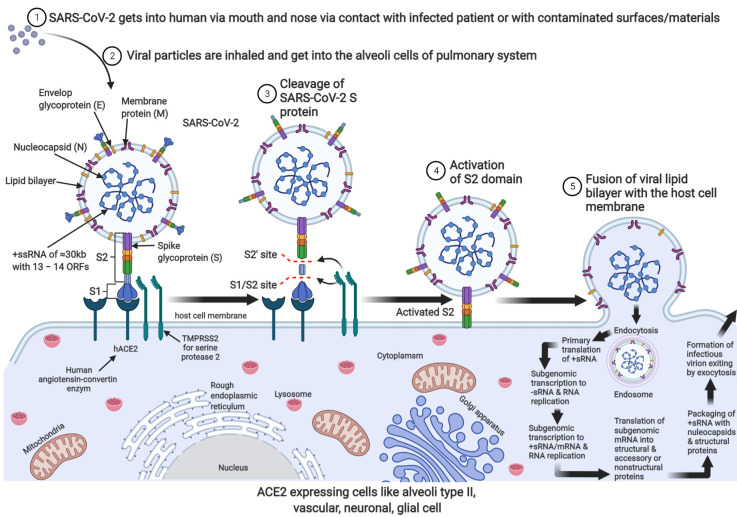
Mechanism of SARS-CoV-2 viral entry, endocytosis, translation, replication, viral packaging, and exocytosis in ACE2-expressing cells. A complete infectious SARS-CoV-2 is +ssRNA enclosed nucleocapsid surrounded by a lipid bilayer that harbours envelop glycoprotein, a membrane protein with protruding crownlike spike protein, which binds the membrane-located ACE2 enzyme that facilitates proteolytic cleavage of S-protein on the viral particle (2–3). Activated S-protein induces invagination of the host cell membrane leading to endocytic movement of the viral particle (4–5). The endosome is degraded with the help of lysosomal cathepsin L/B to release the viral genome for primary translation to produce the RNA-dependent RNA polymerase (RdRp complex). The polymerase transcribes the +sRNA to sub-genomic s–RNA and subsequently full-length +sRNA and some sub-genomic mRNAs that are translated by the host cell ribosomal machinery into nucleocapsid structural and accessory protein. Successful translation, transcription, and RNA replication lead to viral packaging, and the infectious virion can leave the cell by exocytosis to infect the next cell.

As described in Figure 1, SARS-CoV-2 approaches hACE2 through the S2 domain of the spike glycoprotein, exposing the Furin-like bridge between the S1–S2 protein to transmembrane serine protease (TMPRSS2) and subsequently activates the S2 domain for membrane fusion and endocytosis [46]. There is speculation that proteolytic cleavage of SARS-CoV-2 S-protein creates an unstable receptor binding domain of the viral S-glycoprotein that quickly identify their host ACE2 membrane receptor for membrane fusion and subsequent endocytosis [47,48].

The resultant infection may lead to an infected individual experiencing loss of smell and taste, anxiety, dizziness, depression, aggression, confusion, ataxia, seizures, Guillain–Barré syndrome, meningoencephalitis, acute ischemia, haemorrhagic stroke, and respiratory failure [11,16,43,49,50,51,52,53,54,55,56,57].

It is also plausible that macrophages could carry the virus across the BBB. Here, the virus could attack the endothelial cells causing the BBB damage via the intracranial cytokine storm/infiltration [58,59]. The cytokine storm comes from systemic inflammation or direct attack on the T-lymphocytes and macrophages [60,61]. The subgroups of macrophages express ACE2, such as the cluster of differentiation 68 (CD68^+^) and CD169^+^, which may act as Trojan horses carrying the viral particles across the BBB via transcytosis. This event has been linked to the viral spread, tissue inflammation, and lymphopenia experienced during COVID-19 infection [62]. The evidence of the virus in the cerebrospinal fluid (CSF) and the brain region showed that viral invasion is possible; therefore, inter-synaptic CNS viral invasion cannot be ruled out.

## 3. Possible Role of Cryptococcal Proteases in Promoting Brain Invasion in the Context of Co-Infection with SARS-COV-2

Proteases perform multiple roles in the normal immune response to tissue injury or microbial colonisation. For example, host-derived proteases assist in the recruitment of immune cells to infection sites by deteriorating the basement of membranes [63]. The endothelial barrier breakdown creates a passage that allows immune cells to migrate from the blood to reach the inflamed tissue. However, if the secretion of these enzymes is not regulated within an organism, it could impair physiological homeostasis [64]. In turn, this might unintentionally promote microbial pathogenesis by organisms such as *Cryptococcus neoformans*, which operate optimally at the apex of a proteolytic storm. Typically, in such a scenario, the presence of invading microbes induces the production of pro-inflammatory cytokines, which at the same time increases the levels of proteases [65]. In addition to this, Elkington argued that such pathogens could also purposefully secrete microbial proteases to skew the immune response towards tissue damage to promote invasion (Table 2). There are a number of proteases, which are classified according to their catalytic mechanism—based on the configuration of the active site [66], and the reader may peruse several databases that provide information on proteases, such as MEROPS (https://www.ebi.ac.uk/merops, accessed on 28 June 2021), Degradome (http://degradome.uniovi.es, accessed on 1 July 2021) and Proteolysis map (https://web.archive.org/web/20170420071011/http://sonny.burnham.org/proteases, accessed on 1 July 2021) [67,68,69]. Of interest herein is the cryptococcal serine-based protease, metalloprotease (fungalysin, Mpr1) and aspartyl peptidases (May1).

The fungalysin and a closely related bacterial M4 family of proteases called thermolysin share a conserved Pfam domain called FTP (fungalysin/thermolysin propeptide) in their propeptides. They display a domain organisation that has a large pro-domain with an activation locus, a catalytic domain with a zinc-binding site, and a C-terminal hemopexin domain [64,70]. Kryštůfek et al. [71] reported that homology modelling predicted that the May1 adopts a renin fold, which is a predominantly β-sheet conformation characteristic of the aspartyl proteinase family. The substrate-binding cleft and the active site are at the junction of two structurally similar domains of approximately equal size. Moreover, the catalytic residues are located centrally in the cleft with the carboxyl side chains and surrounding main chain scaffolding related by an approximate twofold interdomain axis [71]. The cryptococcal serine-based protease(s) are, at present, unnamed [72]. It is plausible that, like other serine-based proteases, this cryptococcal protease is similarly organised with serine as the nucleophilic amino acid in the active site. It is unknown if this protease is trypsin-like or subtilisin-like.

Often when invading cryptococcal cells in the lung space are not arrested, they can disseminate via a haematogenous route to invade every organ of the body [73]. However, cells have a particular predilection for the brain. For cryptococcal cells to reach the brain, they have to cross the blood–brain barrier (BBB) to cause a fatal infection called meningitis [74,75]. To achieve this, cells can traverse between the damaged tight junctions of the BBB via a paracellular passage [76]. Here, the cryptococcal proteases are, in part, instrumental in this regard. These proteins are secreted extracellularly by cryptococcal cells to promote host invasion [77,78,79,80,81].

**Table 2:** Properties of some proteases of interest that may facilitate SARS-CoV-2 brain invasion.

Sequence	Type	Source	MW (kDa)	pH	Catalytic Residue	Zinc Chelating	Traditional Function	Purported Function in the Context of SARS-CoV-2 Infection	References
1	Serine-based protease (uncharacterised)	*Cryptococcus neoformans*	75	7.2	Ser	No	Promotes fungal brain invasion	May activate the viral S protein or promote viral transcellular migration	[72,77,78]
2	Fungalysin (metalloprotease, Mpr1)	*Cryptococcus neoformans*	42	7.5–8.0	Glu, Tyr, Asp	Yes	Promotes fungal brain invasion	May activate the viral S protein or promote viral transcellular migration	[79,80,81]
3	Major aspartyl peptidase (May1)	*Cryptococcus neoformans*	45	5	Asp	No	Promotes fungal brain invasion	May activate the viral S protein or promote viral transcellular migration	[71,82]
4	Furin (serine-based)	*Homo sapiens* (Human)	104	5–8	Asp, His, Ser	No (calcium-chelating)	Activates functionally important protein precursors	Activates the viral S protein for membrane fusion into cells	[83,84,85]
5	Transmembrane serine protease 2 (TMPRSS2) (serine-based)	*Homo sapiens* (Human)	58	8	His, Asp, Ser	No (calcium-chelating)	Promotes spike protein cleavage and activation for membrane fusion and viral uptake	Activates the viral S protein for membrane fusion into cells	[86,87,88]

In the same way, proteolytic activation of SARS-CoV-2 is required prior to membrane fusion and endocytosis (Figure 1). There is evidence that shows that the viral spike protein can be primed for fusion activation by endosomal acidification where cathepsin L resides and proteolytic cleavage by exogenous proteases is 100- to 1000-fold more efficient [89]. Therefore, it is reasonable to suggest that the cryptococcal proteases could activate viral membrane fusion because of the ability of this fungus to thrive in acidic environments. Thus, any inhibitor of endosomal acidification will inhibit the growth of cryptococcal cells, and in turn, limit viral entry. It has been shown that weak acids such as chloroquine and primaquine are able to accumulate inside organelles such as endosomes and macrophages by ion trapping [90,91]. This results in the neutralization of the internal pH. The raised pH is reported to impair the proteolytic action of enzymes and limits the availability of nutrients crucial for the growth and survival of internalised microbes [92,93,94].

**Figure 2:**
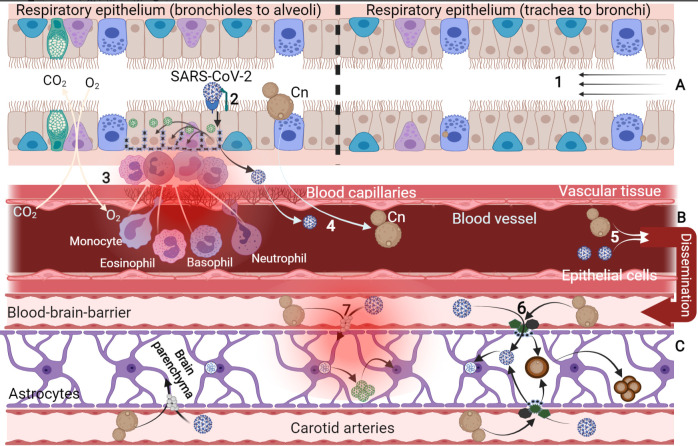
Pulmonary infection caused by *C. neoformans* and SARS-CoV-2 virus. Pulmonary cryptococcosis and severe acute respiratory syndrome caused by coronavirus share some common pathological features. These include targeting alveoli cells, impairing gas exchange mechanism, degrading epithelial surfactant, inducing a cell-mediated immune response, tissues’ invasion and dissemination, systemic inflammation, leucocyte infiltration, extrapulmonary circulation, and brain invasion via carotid arteries (**A**–**C**). (**1**) Air passageway from the upper to the lower respiratory system where infectious fungi (*C. neoformans*) and viral particles enter into the alveoli space. Infectious basidiospores are lodged in the alveoli and find their way across the epithelial tissue. Germinating yeasts are identified by the residential dendritic cells and macrophages that engulf and destroy the yeast pathogen. However, in immunocompromised patients, the pathogenic yeast survives the phagocytic effect and transport as “trojan horse macrophage” across the epithelial tissue into other body organs, including the brain, resulting in cerebral meningoencephalitis; (**2**) SARS-CoV-2 viral particles identify the hACE2 receptor followed by proteolytic cleavage to activate the virus for membrane fusion and endocytosis; (**3**) other epithelial cells are targeted by the replicating virus for destruction, which orchestrates pathological indexes including oxidative damage, mitochondrial degeneration, inflammation, and atrophy, which promote the secretion of pro-inflammatory cytokines and the infiltration of the leucocytes; (**4**) within this event, the viral particles and macrophage-surviving *C. neoformans* find their way into the blood (viraemia and fungaemia, respectively) via the retrograde blood capillaries; (**5**) pathogens end up disseminated into the brain region via carotid arteries and other systemic brain transport arterioles into the medulla and pons region; (**6**) at the BBB, *C. neoformans* could secrete vesicles of hydrolytic and proteolytic enzyme such as phospholipase, urease, metalloproteinase, and hyaluronic acid to enhance transcellular crossing into the brain parenchyma. Compromising the tight junction this way could also enhance intracranial invasion of the viral particles. Again, within the brain region, *C. neoformans* can synthesis melanin due to abundant DOPA substrates, form titan cells, and aggregate to colonise; (**7**) by way of hypothesis, extracellular protease of *C. neoformans* could possibly activate the virus S-protein to promote membrane fusion and endocytosis into the brain neuroglia and neurons via the retrograde nerve ending (dendrites). Viral genome translation, transcription, packaging, and replication in the brain cells lead to neuropathy and viral colonisation in the brain medulla and pons. This leads to neuronal dysfunctions, pathologically characterised by encephalitis, inflammation, cytokine flooding, demyelination, impaired breathing, cardiac rate, muscle contraction, and blood pressure.

By speculation, we envisaged that any pathological condition that weakens the blood–brain barrier (BBB) and brain endothelial blood vessels would increase intracranial invasion of the viral particles via transcellular migration (Figure 2). Therefore, by necessary implication, this may also be true for other protease-secreting microbes. The above discussion shows that the production of proteases may be an ideal target for drug development or repositioning because of their precise and impeccable roles in activating pathogen-related processes.

Apart from the potential roles of microbial proteases to activate or aid in the tissue invasion of the SARS-CoV2 virus, human tissues host a vast majority of these proteases, which include secreted proteases (trypsin, chymotrypsin, renin, elastin, kallikrein, plasmin), membrane-surface expressed proteases such as ACE, compartmentalised proteases including the cathepsin family (B, G, H, and L), lysosyme, ribozyme, and nucleoproteases. Some are pH-dependent (such as cathepsin), metal-dependent (such as metalloproteinases), or position-dependent (such as carboxypeptidase, endo- and exo-peptidases). Based on the catalytic site of each of these enzymes, they are further classified as serine-, threonine-, cysteine-, and aspartyl-proteases. The majority of the host secreted proteases are promiscuous in their activities [95]; therefore, their actions have to be guided by the systemic activation–deactivation cycles. Surface receptor expressed protease (like ACE), ribozyme, and nucleoproteinase are very specific and only respond to inducers, activators, or repressors.

Regarding the activation of SARS-CoV-2 viral particle, ACE-2 exquisitely exhibits specific activity to this receptor binding-pseudo-substrate due to the presence of receptor- binding domain on the SARS-CoV-2 S1 spike protein, which eventually exposes the Furin-like linkage between the S1 and S2 domains of the viral spike glycoprotein. This single activation initiates the endocytotic movement of the viral particles [96]. In addition to this serine-protease carboxypeptidase, cathepsin is another pH-dependent C1-family member of papain-like cysteine protease that is abundant in all cells and can specifically initiate the activation and internalisation of SARS-CoV-2 viral particles, albeit at a lower activity compared to the serine protease. Apart from these two proteases, other host proteases may not activate the SARS-CoV-2 virus. By inference, we proposed that similar serine- or papain-like cysteine protease from infectious bacteria and fungi can co-activate the SARS-CoV-2 viral particle to aggravate the pathological conditions. This may be true if the enzymes share some level of amino acid homology with the host proteases.

Like other enzymes in living systems, the action of proteases is also tightly controlled by inhibitors, which can arrest their catalytic activity. Animal (vertebrate) protease inhibitors are localised in tissues as secretory proteins that block the activity of endogenous and exogenous proteases to prevent unwanted proteolysis [66]. Commercial sources of serine-based protease inhibitors, some of which are biologically extracted and purified, showing specificity towards mammalian serine proteases, may be useful in this regard. In addition to this, the papain-like cysteine protease inhibitors such as leupeptin, E64, E64d, E64c, and cystatin can modify the thiol group at the active site of this enzyme to block its activity. The potencies of these protease inhibitors have been demonstrated to reduce viral antigen titre by targeting cathepsin L (CatL) [97,98]. A vast majority of these inhibitors against the serine, threonine, and cysteine-protease activities and their biological applications have been well documented [99,100].

Concerning SARS-CoV-2, there is evidence that shows that specific protease inhibitors could block the viral cellular entry. In a cell model viral entry system, a commercially available serine protease inhibitor, camostat mesylate, partially blocked the SARS-CoV viral entry [101,102]. However, total inhibition of entry was observed when combined with cathepsin Β/H/L cysteine inhibitor, E-64d (known under different names such as aloxistatin/loxistatin/(2*S*,3*S*)-trans-Epoxysuccinyl-L-leucylamido-3-methylbutane ethyl ester/(1*S*,2*S*)-2-(((*S*)-1-((4-Guanidinobutyl)amino)-4-methyl-1-oxopentan-2-yl)carbamoyl) cyclopropanecarboxylic acid) [102,103].

This E64d is the ethyl ester derivate of E64c, which has gained a lot of attention as a therapeutic agent both in animal and human studies to correct physiological dysfunction, regulated enzyme formation, and prevent viral activation [99,104]. To show the diversity of protease inhibitors, caspases are cysteine-aspartyl-dependent protease whose activity is not inhibited by the epoxysuccinate derivates of E64. However, this enzyme, together with other cysteine proteases such as papain, calpains, caspases, cathepsin B, H, and L, is inhibited by peptidylchloromethylketones—except cathepsin G, which is slowly inhibited [99]. As much as protease inhibitors hold promising therapeutic effects, thorough care should be put in place to weigh the physiological and disease-preventing effects of these inhibitors when applying clinically. For example, E64 and their family derivatives are potent inhibitors of cysteine proteases (cathepsin B, H, L, and calpain), reducing muscular dystrophy, improving locomotor activity, protecting proteoglycan degradation, preventing lesion and apoptosis, attenuating parathyroid hormonal effect on calpain, preventing viral replication and coronavirus protein processing and other biological effects; however, it is associated with hepatic injury, teratogenesis, and attenuated myofibril assembly [99]. The use of protease inhibitors in the treatment of other viral pathogens, such as HIV and hepatitis C, is already established. Unfortunately, treatment can also induce metabolic syndromes such as dyslipidemia [105]. At the moment, Pfizer is exploring the use of a protease inhibitor viz. SARS-CoV-2-3CL, to inhibit viral entry. The drug is reported to have potent in vitro anti-viral activity against SARS-CoV-2 [106]. It will be interesting to observe if the therapeutic benefits of this drug will outweigh its side-effects.

## 4. Conclusions

An understanding of how the priming of the spike protein occurs is fundamental to understanding viral pathogenesis. We speculate that the regulation of protease levels via inhibiting the growth of protease-secreting microbes may contribute to the impairment of host invasion by SARS-CoV-2. Therefore, there is a need to accurately diagnose and resolve underlying microbial infections, which may exogenously avail proteases within an afflicted host—and unintentionally promote SARS-CoV-2 invasion. The latter is also applicable to all infectious members of the β-coronavirus genus that could cause pathological conditions in people with underlying microbial infections.

## Figures and Tables

**Table 1:** The disproportionate reports concerning the co-occurrence of SARS-CoV-2 with non-infectious compared with infectious conditions.

The Co-Occurrence of SARS-CoV-2 with Other Infections					
Non-Infectious Conditions			Aetiological Agents		
	Scopus	PubMed		Scopus	PubMed
			Meningitis-causing agents		
Cancer	42,719	12,180	*M. tuberculosis*	8440	1206
Diabetes	26,288	6663	*S. pneumoniae*	1065	122
Hypertension	16,623	4393	*P. aeruginosa*	1773	107
Obesity	14,643	2923	*H. capsulatum*	49	8
Cerebrovascular disease	4351	1628	*C. neoformans*	160	11
Asthma	8238	1358	*Mucoralean* spp.	4	26
			Non-meningitis-causing agents		
Renal failure	6098	1656	*A. fumigatus*	435	44
Chronic obstructive pulmonary disease	4613	779	*Ca. albicans*	840	36
Hepatic dysfunction	1698	1956	*Ca. auris*	187	107

The data was generated by searching Scopus (period: 2020–2021) and Pubmed (period: 2020–2021) using “SARS-CoV-2” and a “specific non-infectious condition” or a “specific aetiological agent”. *M.* = *Mycobacterium*; *S.* = *Streptococcus*; *P.* = *Pseudomonas*; *A.* = *Aspergillus*; Ca. = *Candida*; *C.* = *Cryptococcus*; *H.* = *Histoplasma*.

## Data Availability

The data presented in this study are available in the article.
